# Polymorphisms in dopaminergic system genes; association with criminal behavior and self-reported aggression in violent prison inmates from Pakistan

**DOI:** 10.1371/journal.pone.0173571

**Published:** 2017-06-05

**Authors:** Muhammad Imran Qadeer, Ali Amar, J. John Mann, Shahida Hasnain

**Affiliations:** 1 Department of Microbiology and Molecular Genetics, University of the Punjab, Lahore, Pakistan; 2 Department of Human Genetics and Molecular Biology, University of Health Sciences, Lahore, Pakistan; 3 New York State Psychiatric Institute, NY and Department of Psychiatry, Columbia University, New York, United States of America; 4 The Women University Multan, Multan, Pakistan; Hudson Institute, AUSTRALIA

## Abstract

Genetic factors contribute to antisocial and criminal behavior. Dopamine transporter *DAT-1* (SLC6A3) and *DRD2* gene for the dopamine-2 receptor are dopaminergic system genes that regulate dopamine reuptake and signaling, and may be part of the pathogenesis of psychiatric disorders including antisocial behaviors and traits. No previous studies have analyzed *DAT-1* and *DRD2* polymorphisms in convicted murderers, particularly from Indian subcontinent. In this study we investigated the association of 40 bp VNTR polymorphism of *DAT-1* and Taq1 variant of *DRD2* gene (rs1800479) with criminal behavior and self-reported aggression in 729 subjects, including 370 men in Pakistani prisons convicted of first degree murder(s) and 359 control men without any history of violence or criminal tendency. The 9R allele of *DAT-1* VNTR polymorphism was more prevalent in convicted murderers compared with control samples, for either one or two risk alleles (OR = 1.49 and 3.99 respectively, P = 0.003). This potential association of *DAT-1* 9R allele polymorphism with murderer phenotype was confirmed assuming different genetic models of inheritance. However, no genetic association was found for *DRD2* Taq1 polymorphism. In addition, a combined haplotype (9R-A2) of DAT-1 and DRD2 genes was associated with this murderer phenotype. Further, 9R allele of *DAT-1* was also associated with response to verbal abuse and parental marital complications, but not with other measures pertinent to self-reported aggression. These results suggest that 9R allele, which may influence levels of intra-synaptic dopamine in the brain, may contribute to criminal tendency in this sample of violent murderers of Pakistani origin. Future studies are needed to replicate this finding in other populations of murderers and see if this finding extends to other forms of violence and lesser degrees of aggression.

## Introduction

Antisocial delinquency and criminal behavior is a serious social concern in most eastern and western societies [1]. The adverse effects of antisocial and criminal behavior involve individual life, family and wider society [2]. Criminal delinquency include disturbed family relations, substance use disorders, underachievement in educational and professional realms and continued involvement in violent criminal acts with resultant suffering of the victims of these criminal acts, their families and communities [3, 4].

Some of the genetic and environmental antecedents of antisocial and criminal behavior have been identified [5]. Shared environmental influences are also important factors governing criminal behavior [6]. The heritability of antisocial behavioral phenotypes, including criminal behavior and human noncriminal aggression, has been estimated to be approximately 50% [7–11].

Genes involved in dopaminergic neurotransmission, such as those expressing the dopamine transporter and dopamine-2 receptor, are promising candidate genes for antisocial behaviors and traits [12–14]. The polymorphisms in dopaminergic genes have been associated with psychiatric and developmental disorders like ADHD and autism, personality traits and substance use disorders. Moreover, the dopaminergic antagonists are pharmacotherapeutic employed for human aggressive behavior [15]. However, apart from the reports of Guo et al, the studies investigating the role of dopaminergic gene variants in modulation of more extreme phenotypes of violent or homicidal behavior have been scarce [14].

Two of the best studied polymorphisms of dopaminergic system are dopamine transporter (*DAT-1*) variable number of tandem repeats (VNTR) and TaqI A polymorphism of dopamine receptor D2 (DRD2) gene [37]. *DAT-1* (also known as *SLC6A3*) maps to short arm of chromosome 5 at 5p15.3 and codes for the dopamine transporter protein which regulates the level of intra-synaptic dopamine and thereby dopamine receptor activation [16]. It does so by reuptake released dopamine back into the presynaptic terminal.

The 3’ noncoding region of *DAT-1* contains a 40 bp VNTR sequence that can be present in 3–11 copies. The 9R and 10R variants of this functional VNTR are the most common ones in different populations [17]. The *DAT-1* VNTR polymorphism is a functional polymorphism affecting dopamine transport protein expression [18, 19]. Which allele of the *DAT-1* VNTR polymorphism (9R vs 10R) mediates the most appropriate gene expression remains uncertain [20]. The *DAT-1* 40 bp VNTR polymorphism has been found to be associated with violent delinquency [14], externalizing problem behavior [13, 21], neuropsychiatric disorders such as Tourette syndrome [22], ADHD [23, 24], schizophrenia [25], psychoactive substance abuse [26, 27] and reward sensitivity [28].

The Taq1A single nucleotide polymorphism (SNP) in the *DRD2* gene arises from a T to C transition at a site located approximately 10 Kb downstream from the end of the gene. The Taq1 polymorphism affects D2 receptor activity and density in some studies [29, 30] but not others [31]. The *DRD2* Taq1 SNP was demonstrated to be associated with serious and violent delinquency in adolescents and young adults [14], as well as substance use disorder [32, 33] and alcoholism [34].

Based on the potential involvement of dopaminergic system genes in susceptibility to aggression and criminal behavior, we conducted this study on Pakistani jail inmates convicted for murder as an extreme phenotype in order to determine if the VNTR polymorphism in *DAT-1* and Taq1 SNP of *DRD2* genes were associated with serious criminal behavior and self-reported aggression.

## Material and methods

### Consent

This study was performed after obtaining due permission from higher police authorities as it involved jail inmates as subjects. Written informed consent was obtained from all study participants. The study was approved by Institutional Ethics Committee at University of the Punjab, Lahore, which adheres to the declaration of Helsinki.

### Subjects

The present study included 370 men imprisoned in three major district jails in the province of Punjab because of conviction for single or multiple homicides. An effort was made to recruit only those individuals who have been convicted of the crime based on either forensic evidence or alleged/condemned status. After obtaining informed written consent, the subjects were interviewed to access the self-reported aggression using a comprehensive questionnaire based on State Trait Anger Expression Inventory (STAXI) and blood samples were collected for genetic analysis. All the subjects belonged to three major populations of Punjab with similar linguistic affiliations. The control group of 359 subjects was selected from the same three populations in a similar ratio to cases in order to match the ethnicity. The individuals included in the control group had no history of violent behavior or criminal activities, based on social records and information obtained from other family members of the individuals.

### DNA extraction and genotyping of *DAT-1* and *DRD2* polymorphisms

Genomic DNA was isolated from EDTA-anticoagulated blood samples using a modification of standard phenol-chloroform extraction method [35]. The 40 bp 3’ VNTR polymorphism of the *DAT-1* gene was genotyped as described by Vandenbergh et al. with modifications [36]. The primer pair: Forward, 5’-TGCGGTGTAGGGAACGGCCTGAG-3’ and Reverse, 5’-CTTCCTGGAGGTCACGGCTCAAGG-3’ were used to amplify a polymerase chain reaction (PCR) product that was 440 bp and 480 bp for the two most common 9R allele and 10R allele, respectively. The PCR products were run on a 3% agarose gel and genotypes were determined for each sample.

All subjects were genotyped for the TaqI A marker at the dopamine D2 receptor locus using a PCR-RFLP based approach, as described by Grandy et al [37]. Specific primer set; Forward, 5’-GACATGATGCCCGCTTT-3’ and Reverse, 5’-TCATCAACCTCCTAGAACAT-3’ were used to amplify a 199 bp fragment PCR. The PCR products were subjected to digestion with TaqI A restriction endonuclease for 12 hours at 65 C and digested fragments were resolved on a 3% agarose gel to determine the respective genotypes for each sample. The genotypes were scored by two independent individuals, in order to improve reliability of genotype calls.

### Statistical analysis

Data pertaining to basic characteristics of the subjects was coded and analyzed using the Statistical Package for Social Sciences (SPSS) version 20 for Windows. Allele and genotype frequencies are represented as numbers (percentage), which were determined by direct counting and compared between groups using Chi square test. Conformance of genotype frequencies to Hardy-Weinberg equilibrium was tested using the Chi square test. Odds ratio (OR) with 95% confidence interval (CI) and associated p-value was calculated as a measure of association using SNPstats program [38]. Combined genotype frequencies for *DAT-1* and *DRD2* genes were also determined and compared using the same SNPstats program. A two tailed p value of less than 0.05 was considered significant unless otherwise stated.

## Results

### Basic characteristics of subjects

This study included 729 men, comprised of 370 prison inmates convicted of first-degree murder(s) and 359 individuals in a control group with no history of violent or criminal acts. The controls were matched with cases on demographic details and the two groups did not differ with respect to age (*p* value 0.177), district (*p* value 0.456), socio-economic status (*p* value 0.677) and education level (*p* value 0.296). The basic characteristics of subjects, including parameters used as measure of self-reported aggression, are presented in S1 Table. The complete data pertaining to this study are available as S1 Original Dataset in the supporting information.

### Allele, genotype frequencies and genetic associations

The basic information and allele frequencies observed in prison inmates and controls, for the *DAT-1* and *DRD2* polymorphisms are described in the Table 1. The allelic and genotypic distribution for both, *DAT-1* and *DRD2* polymorphisms analyzed did not deviate from Hardy-Weinberg equilibrium (*p*-values of 0.27 and 0.40 for DAT-1 and DRD2 polymorphisms, respectively).

**Table 1:** Basic information and HWE analysis for studied polymorphisms.

Polymorphism	Gene	Position	Region	Allele	MAF		*p*-value for HWE ^a^	Samples genotyped (%)
					Cases (n = 370)	Controls (n = 359)		
**40 bp VNTR polymorphism**	*DAT-1*	rs28363170 (insertion)	3’UTR	9R	0.21	0.15	0.27	91
**TaqI A SNP**	*DRD2*	rs1800479	10 kb downstream	A1	0.27	0.25	0.40	98

HWE, Hardy–Weinberg equilibrium; MAF, minor allele frequencies; SNP, single-nucleotide polymorphism; UTR, untranslated region; VNTR, variable number of tandem repeats

^a^ HWE *p*-value in the control group.

The allele and genotype distribution in subjects and controls for *DAT-1* VNTR and *DRD2* TaqI A polymorphisms were compared (Table 2). The 9R allele as well as 9R/9R genotype of *DAT-1* VNTR were more common in the prison inmate group as compared with the control group (OR = 1.57, *p* = 0.002 and OR = 3.99, *p* = 0.003, respectively). However, the allele and genotype distribution for *DRD2* TaqI A polymorphism was comparable in murderers and normal controls.

**Table 2:** Allele and genotype distribution for *DAT-1* and *DRD2* polymorphisms and association with homicide history.

Polymorphisms	Genotype/Allele	Cases n = 370, (%)	Controls n = 359, (%)	OR (95% CI)	*p*-value
*DAT-1* 40 bp VNTR	10R/10R	206 (61.5%)	235 (71.9%)	1 (Referent)	**0.003**
	10R/9R	115 (34.3%)	88 (26.9%)	**1.49 (1.07–2.08)**	
	9R/9R	14 (4.2%)	4 (1.2%)	**3.99 (1.29–12.32)**	
	10R	527 (79%)	558 (85%)	1 (Referent)	**0.002**
	9R	143 (21%)	96 (15%)	**1.57 (1.18–2.09)**	
*DRD2* TaqI A SNP	A2/A2	200 (54.8%)	191 (54.7%)	1 (Referent)	0.341
	A2/A1	135 (37%)	139 (39.8%)	0.93 (0.68–1.26)	
	A1/A1	30 (8.2%)	19 (5.4%)	1.51 (0.82–2.77)	
	A2	535 (73.3%)	521 (74.6%)	1 (Referent)	0.602
	A1	195 (26.7%)	177 (25.4%)	1.07 (0.84–1.35)	

OR, odds ratio; CI, confidence interval; n (%), frequency.

Association of *DAT-1* 40 bp VNTR and *DRD2* TaqI A polymorphisms with history of murder was also analyzed considering different genetic models (Table 3). The *DAT-1* VNTR polymorphism was associated with murder history in all genetic model considered. As indicated by Akaike information criterion (AIC) value, the best predictive model for this association was the one for dominant mode of inheritance, where presence of 9R allele of *DAT-1* VNTR polymorphism, in either homozygous or heterozygous form, conferred an increased risk of murder history (OR = 1.60, *p*-value 0.0046). However, the TaqI A SNP of *DRD2* failed to reveal any association under all tested genetic models.

**Table 3:** Association of the studied polymorphisms with criminal delinquency assuming different genetic models.

Polymorphisms	Model	Genotypes	Cases, n (%)	Controls, n (%)	OR (95% CI)	*p*-value
***DAT-1* 40 bp VNTR**	Dominant	10R/10R	206 (61.5%)	235 (71.9%)	1.00	**0.0046**
		10R/9R-9R/9R	129 (38.5%)	92 (28.1%)	**1.60 (1.15–2.22)**	
	Recessive	10R/10R-10R/9R	321 (95.8%)	323 (98.8%)	1.00	**0.016**
		9R/9R	14 (4.2%)	4 (1.2%)	**3.52 (1.15–10.81)**	
	Overdominant	10R/10R-9R/9R	220 (65.7%)	239 (73.1%)	1.00	**0.038**
		10R/9R	115 (34.3%)	88 (26.9%)	**1.42 (1.02–1.98)**	
	Log-additive	-	-	-	**1.61 (1.20–2.16)**	**0.0013**
***DRD2* TaqI A SNP**	Dominant	A2/A2	200 (54.8%)	191 (54.7%)	1.00	0.99
		A2/A1-A1/A1	165 (45.2%)	158 (45.3%)	1.00 (0.74–1.34)	
	Recessive	A2/A2-A2/A1	335 (91.8%)	330 (94.6%)	1.00	0.14
		A1/A1	30 (8.2%)	19 (5.4%)	1.56 (0.86–2.82)	
	Overdominant	A2/A2-A1/A1	230 (63%)	210 (60.2%)	1.00	0.44
		A2/A1	135 (37%)	139 (39.8%)	0.89 (0.66–1.20)	
	Log-additive	-	-	-	1.07 (0.85–1.36)	0.56

OR, odds ratio; CI, confidence interval; n (%), frequency

Boldface indicates *p* < 0.05 was considered as statistically significant.

### Combined genotype analysis

In order to analyze the collective effect of *DAT-1* and *DRD2* polymorphisms, the possible combinations of alleles or combined genotype frequencies were also determined as presented in Table 4. The 9R-A2 allelic combination was found to be more frequent in the prison subjects group as compared to the control group (OR = 2.00, p = < 0.0001.

**Table 4:** Distribution of combined genotype frequencies and their association with homicide.

Combined genotypes		Combined genotype frequencies		OR (95% CI)	*p*-value
*DAT-1*	*DRD2*	Cases	Controls		
10R	A2	0.566	0.649	1 (Referent)	-
10R	A1	0.220	0.203	1.23 (0.92–1.65)	0.16
9R	A2	0.1667	0.0971	**2.00 (1.34–2.98)**	**< .0001**
9R	A1	0.0472	0.0499	1.12 (0.60–2.11)	0.71

OR–odds ratio; 95% CI– 95% confidence interval. Boldface indicates p < 0.05 was considered as statistically significant.

The polymorphism data for *DAT-1* VNTR and *DRD2* TaqI A polymorphisms were also evaluated after dividing the prison inmates into sub-groups manifesting self-reported aggression and histories such as response to physical or verbal abuse, history of parental aggression, or childhood and substance use disorder (Table 5). However, no significant differences were observed in any of the comparisons made except for aggressive response to verbal abuse and parental marital complications where prison inmates carrying the 9R allele of *DAT-1* polymorphism reported being more likely to be provoked by verbal abuse and having experienced parental marital complications than 9R non-carriers (OR = 1.85, p-value 0.025 and OR = 1.73, p-value 0.041, respectively).

**Table 5:** Association of *DAT-1* and *DRD2* polymorphisms with self-reported aggression and histories.

Measure	*DAT-1* genotypes (Dominant model)		OR (95% CI)	*p*-value	*DRD2* genotypes (Recessive model)		OR (95% CI)	*p*-value
	10R/10R (n = 206)	10R/9R+9R/9R (n = 129)			A2/A2+A2/A1 (n = 335)	A1/A1 (n = 30)		
**Self-reported aggression (Lifetime)**								
Yes	133 (66.2%)	82 (66.7%)	1.02 (0.63–1.64)	1	223 (68.6%)	18 (64.2%)	0.82 (0.36–1.84)	0.791
No	68 (33.8%)	41 (33.3%)			102 (31.4%)	10 (35.7%)		
**Provoked by verbal abuse**								
Yes	110 (59.1%)	78 (72.9%)	**1.85** **(1.10–3.11)**	**0.025**	186 (64.1%)	16 (57.1%)	0.74 (0.33–1.63)	0.596
No	76 (40.9%)	29 (27.1%)			104 (35.9%)	12 (42.9%)		
**Provoked by physical abuse**								
Yes	109 (58.3%)	73 (68.2%)	1.53 (0.93–2.53)	0.118	181 (62.2%)	16 (57.1%)	0.81 (0.36–1.77)	0.751
No	78 (41.7%)	34 (31.8%)			110 (37.8%)	12 (42.9%)		
**Childhood history of abuse**								
Yes	132 (65.7%)	78 (62.4%)	0.86 (0.54–1.38)	0.631	208 (63.8%)	20 (66.7%)	1.13 (0.51–2.50)	0.92
No	69 (34.3%)	47 (37.6%)			118 (36.2%)	10 (33.3%)		
**History of parental aggression towards future aggressor (murderer)**								
Yes	102 (50.5%)	64 (51.6%)	1.04 (0.66–1.63)	0.920	172 (52.6%)	9 (32.1%)	0.42 (0.18–0.97)	0.059
No	100 (49.5%)	60 (48.5%)			155 (47.4%)	19 (67.9%)		
**Parental marital problems (including divorced and separated with step parents)**								
Yes	44 (21.6%)	41 (32.3%)	**1.73** **(1.05–2.86)**	**0.041**	85 (25.7%)	07 (23.3%)	0.88 (0.36–2.12)	1.00
No	160 (78.4%)	86 (67.7%)			246 (74.3%)	23 (76.7%)		
**Psychiatric illness**								
Yes	10 (5.5%)	4 (3.7%)	0.65 (0.20–2.14)	0.671	17 (5.8%)	0 (0%)	-	0.238
No	172 (94.5%)	105 (96.3%)			275 (94.2%)	25 (100%)		
**Substance use disorder**								
Yes	43 (22.1%)	22 (18.2%)	0.78 (0.44–1.39)	0.492	62 (19.6%)	7 (26.9%)	1.50 (0.60–3.74)	0.521
No	152 (77.9%)	99 (81.8%)			254 (80.4%)	19 (73.1%)		

OR–odds ratio; 95% CI– 95% confidence interval. Boldface indicates p < 0.05 was considered as statistically significant.

## Discussion

In the present study of dopaminergic polymorphisms in prison inmates convicted for murder, we found a significant difference in the frequency distribution of *DAT-1* VNTR alleles in violent criminals and control men of Pakistani origin. The 9R allele of *DAT-1* 3’ VNTR polymorphism, either in homozygous or heterozygous form, was more prevalent in murderers than in nonaggressive control subjects. This potential association of 9R allele of *DAT-1* polymorphism with murder history persisted when assuming different genetic models such as dominant and recessive models as well as in haplotype analysis. Further, 9R of *DAT-1* was also associated with response to verbal abuse and parental marital complications and trend for association with physical abuse but not with drug use disorders including alcoholism. Based upon these findings, we suggest that 9R allele, which may influence the levels of dopamine in these criminals, might be one of the predisposing factors associated with extreme criminal behavior.

Although, none of the previous studies have analyzed *DAT-1* VNTR in convicted murderers, several studies have shown the *DAT-1* VNTR polymorphism to be associated with externalizing behavior and violent delinquency [13, 14, 21]. Consistent with our results, Young et al., found the 9R allele of *DAT-1* polymorphism to be a significant risk factor associated with externalizing behavior in a community sample of young children [21]. Similarly, the minor 9R allele of the *DAT-1* VNTR was implicated as risk allele in cocaine intoxication [39] and alcohol dependence [40, 41]. In contrast, other studies have reported the association of more common 10R allele of *DAT-1* VNTR with ADHD [23, 24, 42] with serious and violent delinquency [14]. A similar study evaluating the role of same *DAT-1* VNTR in externalizing behavior and temperament problems did not detect an association in a general population sample [43].

The disagreement about association of *DAT-1* polymorphism with disruptive behavior may have resulted from differences in measures of antisocial behavior (hyperactivity *vs* externalizing problems *vs* violent delinquency *vs* criminal behavior or simply the severity of criminal behavior or the difference between reactive aggression and pro-active or pre-meditated aggression, some of which are correlated but all are distinct aspects of antisocial or pathological behavior. Moreover, it remains unclear which allele of the *DAT-1* VNTR polymorphism (9R vs 10R) mediates the most appropriate gene expression [20]. Studies to determine the functional relevance of *DAT-1* VNTR polymorphism in dopamine transport protein expression have produced various results. In some studies the 9R allele of *DAT-1* was associated with greater transcription in a murine and human dopaminergic cell line [18, 44, 45]. In contrast, using a non-human primate cell line, the 10R allele of *DAT-1* mediates higher transcription efficiency than 9R allele [19].

Further, evaluating a single polymorphism in each of two well-known genes of dopaminergic system has limitations. Other functional polymorphisms within the *DAT-1* gene or in an adjacent region could be in linkage disequilibrium with the variants examined in this study. It is plausible that these other variants could be the real mediators of predisposition to criminal behavior. Therefore, replication studies using additional genetic variants within the main dopamine-related genes (*DAT-1*, DRD1, *DRD2* and *DRD4*) need to be undertaken in order to elucidate the role of dopaminergic polymorphisms in commission of criminal delinquency and extreme violence.

The present study, based on the molecular evaluation of sentenced prisoners from the Punjab, Pakistan, where 60% consanguinity exists in the population [46], revealed that both heterozygous and homozygous status of 9R allele of DAT-1 gene were more frequent in murderers than in nonaggressive control subjects.

It would be validating if the results obtained in the present study could be supported by a study of dopamine system function, epigenetic effects and dopaminergic polymorphisms in relation to antisocial and aggressive phenotypes and related endophenotypes.

In conclusion, the 9R allele of *DAT-1* VNTR polymorphism is associated with extreme criminal behavior in a sample of violent male prison inmates convicted of murder. However, antisocial behavior and criminal tendency is also prone to the influence of environmental factors. The association of polymorphisms in dopaminergic genes with antisocial behavior and aggression may help explain the genetic contribution towards serious criminal tendencies. Genetic factors involved in criminal behavior may help to determine part of the biological modulation of normal aggression as well as pathological aggression. In spite of the limitations, e.g., ethical, security and safety of both researcher and sentenced murderer, limitation of time allowed to interview and GWAS studies, it is hoped that these findings may guide development of prevention and therapeutic interventions. Further studies should be undertaken in other populations to delineate the role of 9R allele and 10R allele 0f DAT-1 gene with different grades of severity of aggression and violent crime.

## Supporting information

S1 Table Basic characteristics of study subjects (DOCX)

S1 Original Dataset (XLSX)
