# Supplementary material for: Polymorphisms in dopaminergic system genes; association with criminal behavior and self-reported aggression in violent prison inmates from Pakistan
Source: PLoS One. 2017 Jun 5;12(6):e0173571. doi: 10.1371/journal.pone.0173571 (PMC5459412; doi:10.1371/journal.pone.0173571)
Supplement: S1 Table — (DOCX) [file pone.0173571.s001.docx]

**S1 Table. Basic characteristics of study subjects**

| **Variables** | **Frequency in prison inmates (n = 370)** | **Frequency in controls (n = 359)** | **p-value** |
| --- | --- | --- | --- |
| Age (years)^a^ | 36.4±11.8 | 35.2±10.7 | 0.177 |
| **District of Punjab** |  |  |  |
| Sargodha | 181 (48.8%) | 186 (52.5%) | 0.456 |
| Toba tek singh | 99 (26.7%) | 81 (22.9%) |  |
| Mianwali | 90 (24.3%) | 87 (24.6%) |  |
| **Socio-economic status** |  |  |  |
| Poor | 80 (22.1%) | 79 (22.4%) | 0.677 |
| Middle class | 261 (72.1%) | 247 (70.2%) |  |
| Affluent | 21 (5.8%) | 26 (7.4%) |  |
| **Educational level** |  |  |  |
| Illiterate | 137 (37.4%) | 115 (32.7%) | 0.296 |
| High School education | 215 (58.7%) | 218 (61.9%) |  |
| Graduate and above | 14 (3.8%) | 19 (5.4%) |  |
| **Marital status** |  |  |  |
| Unmarried | 150 (40.9%) | - |  |
| Single marriage | 205 (56%) | - |  |
| Complicated | 11 (03%) | - |  |
| **Type of prisoner** |  |  |  |
| Under trial | 87 (23.8%) | - |  |
| Convicted | 25 (6.8%) | - |  |
| Condemned | 253 (69.2%) | - |  |
| **Age at time of committing murder^a^** | 30.4±11.1 | - |  |
| **Murdered or alleged to murder** |  |  |  |
| Yes | 274 (94.5%) | - |  |
| No | 16 (5.5%) | - |  |
| **Motive of murder** |  |  |  |
| Honor | 101 (34.6%) | - |  |
| Property | 56 (19.2%) | - |  |
| Revenge | 134 (45.9%) | - |  |
| Without any reason | 01 (0.3%) | - |  |
| **Murdered by** |  |  |  |
| Gun shots | 243 (81.8%) | - |  |
| Stabbing/execution | 36 (12.1%) | - |  |
| Beating with solid objects | 11 (3.7%) | - |  |
| Strangling | 07 (2.4%) | - |  |
| **Relationship with murdered victims** |  |  |  |
| Blood relative | 117 (39.4%) | - |  |
| From in laws | 22 (7.4%) | - |  |
| Rival | 71 (23.9%) | - |  |
| Stranger | 87 (29.3%) | - |  |
| **Family history of murder** |  |  |  |
| Yes | 72 (24.9%) | - |  |
| No | 216 (75.1%) | - |  |
| **Anyone got murdered in the family** |  |  |  |
| Yes | 70 (24.1%) | - |  |
| No | 221 (75.9%) | - |  |
| **Self-reported aggression** |  |  |  |
| Yes | 242 (67.4%) | - |  |
| No | 116 (32.6%) | - |  |
| **Provoked by physical abuse** |  |  |  |
| Yes | 199 (61.4%) | - |  |
| No | 124 (38.6%) | - |  |
| **Provoked by verbal abuse** |  |  |  |
| Yes | 204 (63.2%) | - |  |
| No | 118 (36.8%) | - |  |
| **Parental marital history** |  |  |  |
| Single marriage | 301 (82.2%) | - |  |
| Complicated | 64 (17.8%) | - |  |
| **Step relations** |  |  |  |
| Yes | 86 (23.7%) | - |  |
| No | 276 (76.3%) | - |  |
| **History of parental aggression** |  |  |  |
| Yes | 185 (51.5%) | - |  |
| No | 175 (48.5%) | - |  |
| **Childhood history of abuse** |  |  |  |
| Yes | 230 (63.8%) | - |  |
| No | 131 (36.2%) | - |  |
| **Any psychiatric illness** |  |  |  |
| Yes | 17 (5.3%) | - |  |
| No | 305 (94.7%) | - |  |
| **Substance use disorder** |  |  |  |
| Yes | 69 (19.8%) | - |  |
| No | 278 (80.2%) | - |  |

^a^ Data is mean ± SD

Note: The total number differs from variable to variable because information was either not available or not provided for that variable
